# Supplementary material for: Building blocks for commodity augmented reality-based molecular visualization and modeling in web browsers
Source: PeerJ Comput Sci. 2020 Feb 17;6:e260. doi: 10.7717/peerj-cs.260 (PMC7924717; doi:10.7717/peerj-cs.260)
Supplement: Supplemental Information 1 [file peerj-cs-06-260-s001.docx]

Supporting Information for

**Building blocks for commodity augmented reality-based molecular visualization and modeling in web browsers**

*Luciano Andrés Abriata*

Laboratory for Biomolecular Modeling, School of Life Sciences, École Polytechnique Fédérale de Lausanne and Swiss Institute of Bioinformatics, CH-1015 Lausanne, Switzerland.


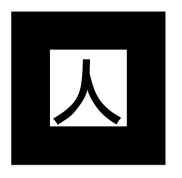

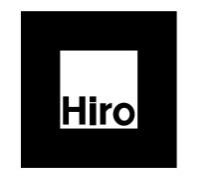

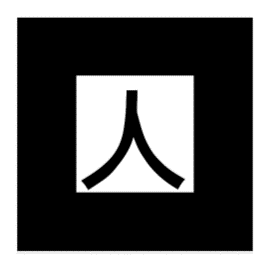

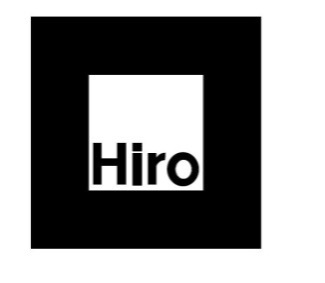

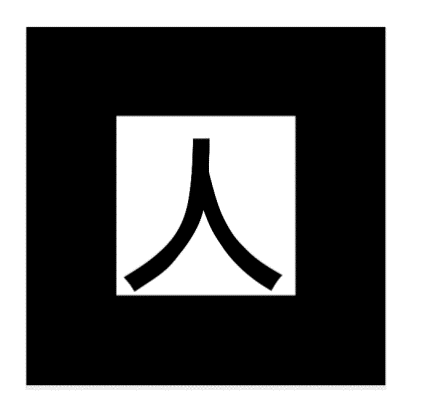


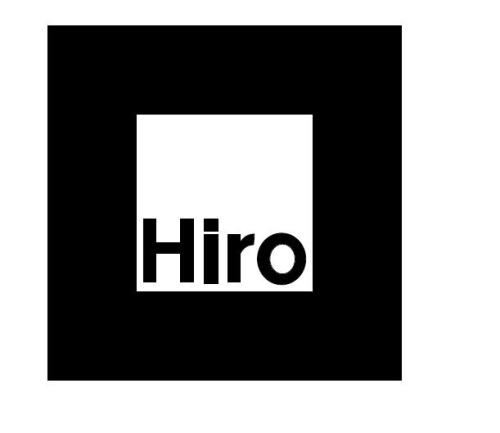

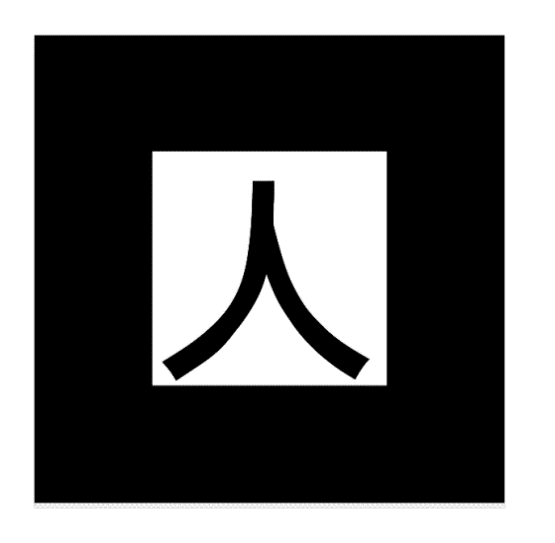

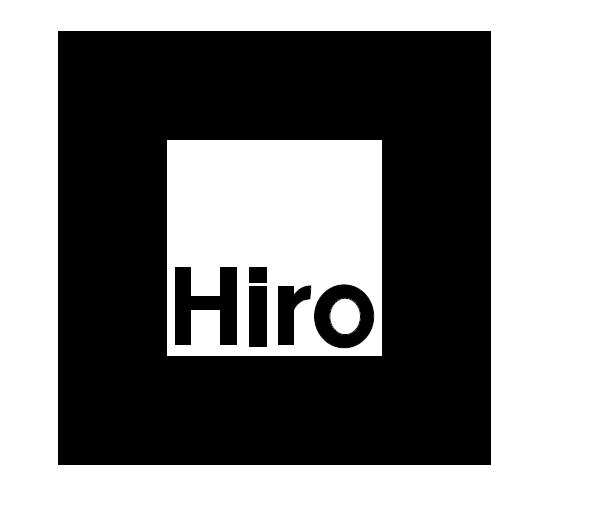


**Figure S1.** The Kanji and Hiro markers laid out in four different sizes, ready to print. It is recommended to glue the used markers on small devices that can be easily handled.


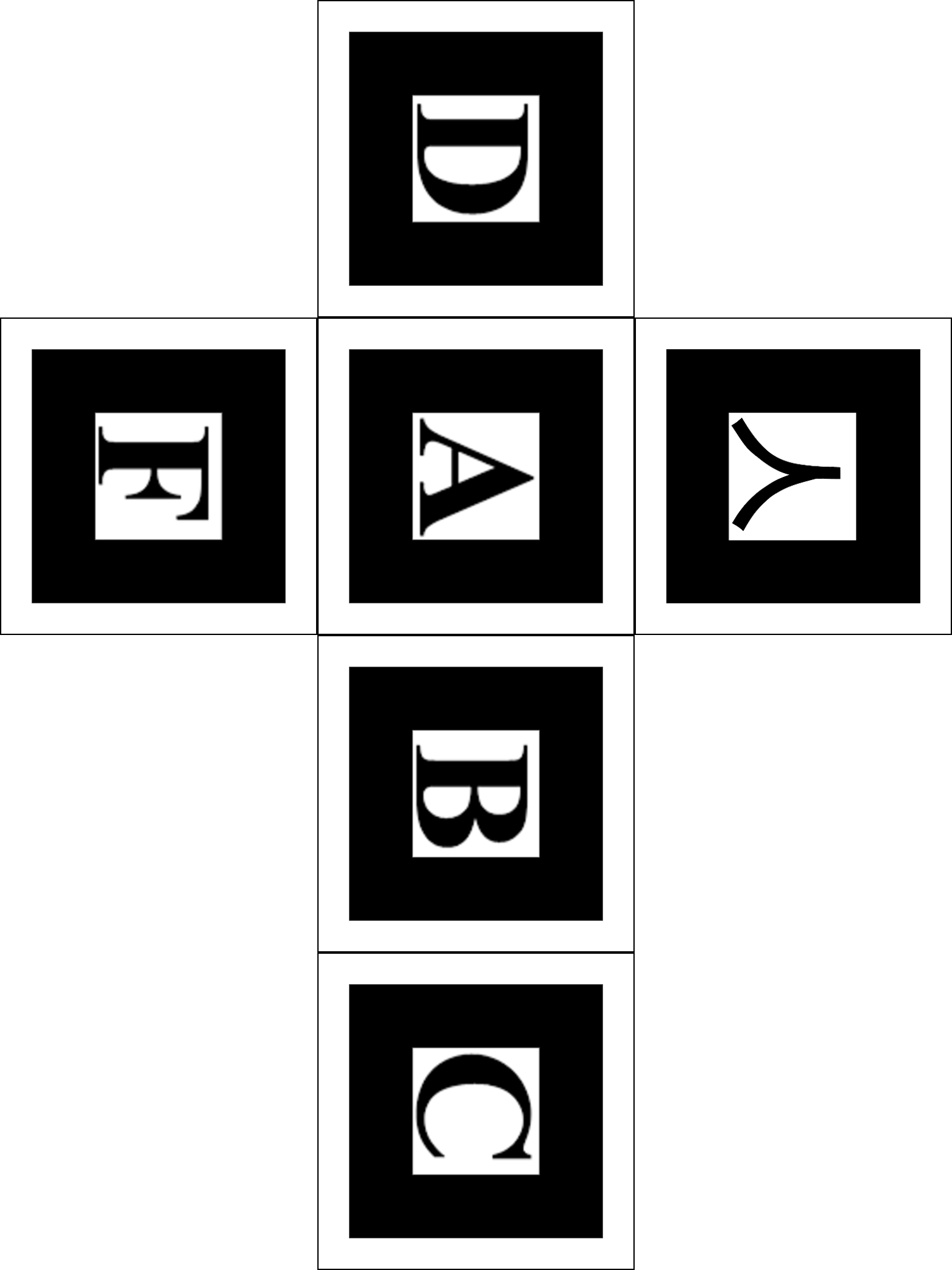


**Figure S2.** An example cubic marker, by Prof. Lee Stemkoski and available at <https://github.com/stemkoski/AR-Examples/blob/master/markers/cube.png>

**
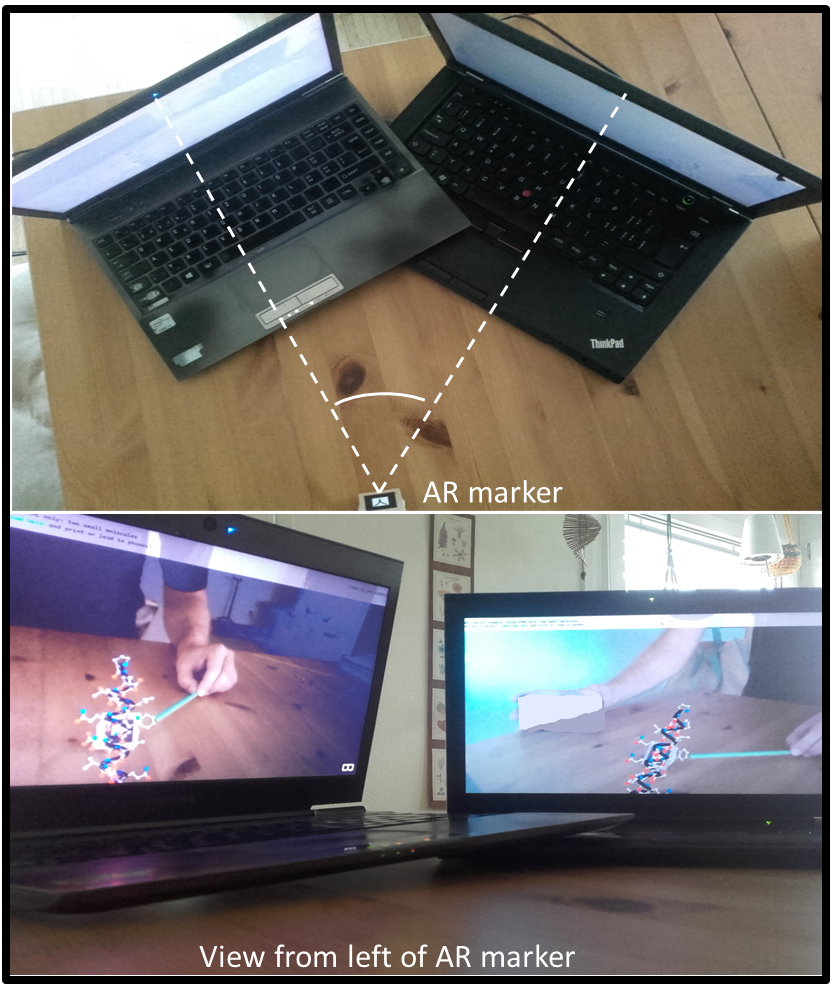
**

**Figure S3.** A small protein helix including an aromatic side chain, displayed on the same physical marker observed from two orientations. A physical green stick points at the aromatic amino acid. The example shows how marker-based AR facilitates concurrent view by multiple users.

**
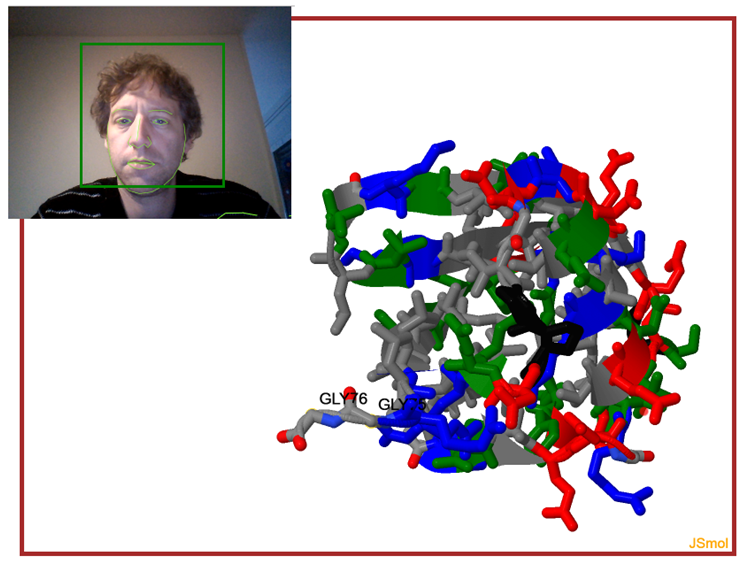
Figure S4.** WebGazer.js put to work to control JSmol, so that whatever region of the protein the user looks at comes to the front. I acknowledge Angel Herráez for help integrating both libraries. Example accessible at <https://lucianoabriata.altervista.org/jsinscience/jsmolwebgazer/jsmolwebgazer.html>.
